# Supplementary material for: Brains over beauty: A preregistered test of the effects of objectification on women’s cognitive performance
Source: PLoS One. 2023 Sep 21;18(9):e0291853. doi: 10.1371/journal.pone.0291853 (PMC10513279; doi:10.1371/journal.pone.0291853)
Supplement: Attachment — Submitted filename: Brains Over Beauty Cover Letter.docx [file pone.0291853.s001.docx]

Dear Dr. Sokolov,

Thank you for the opportunity to revise this manuscript. We appreciate the detailed and thoughtful comments of the three reviewers. Although we were disappointed with Reviewer 1’s conclusions about the merits of this study, we were heartened by the positive responses of Reviewers 2 and 3 and believe we have addressed the concerns of all three reviewers in this revision. In particular, in response to Reviewer 1, we have added substantial detail about the body of published findings that address a potential moderating role of trait self-objectification. In several areas of the paper, we have added more precise details about the specific methodologies used in related research and have attempted to more clearly delineate how our paper fits into this body of research. We have created an updated analysis script (R script) and Bayes analyses output as requested by reviewers and made these available in our [online supplementary materials](https://osf.io/7n34k/?view_only=aeb19dcedac24b0ba8c4998f7a794af6). Our response to each reviewer’s comment is in bold below.

Amended funding statement:

This research was supported by the Benton J. Underwood Summer Research Fellowship awarded by the Northwestern Psychology Department and an Academic Year Research Grant awarded by Northwestern University’s Office of Undergraduate Research (both awarded to AZ). The funders had no role in study design, data collection and analysis, decision to publish, or preparation of the manuscript. There was no additional external funding received for this study.

Sincerely,

Anne Zola, MS

annezola2019@u.northwestern.edu

Senior Research Data Analyst, Medical Social Sciences

Northwestern University

**Response to Editor and Reviewer comments**

Please explain and discuss any differences of your research from the original one by Gay and Castano as mentioned by Reviewer 1.

**We have added additional details in both the introduction and discussion section on the differences between the current study and other studies where participants were videoed (Gay & Castano, 2010) or photographed (Guizzo & Cadinu, 2017) by a male or female experimenter.**

2. Thank you for stating in your Funding Statement:

“This research was supported by grants from the Northwestern University Undergraduate Research Grants Program (awarded to AZ). The funders had no role in study design, data collection and analysis, decision to publish, or preparation of the manuscript.”

Please provide an amended statement that declares *all* the funding or sources of support (whether external or internal to your organization) received during this study, as detailed online in our guide for authors at [http://journals.plos.org/plosone/s/submit-now.](https://urldefense.com/v3/__http:/journals.plos.org/plosone/s/submit-now.__;!!Dq0X2DkFhyF93HkjWTBQKhk!TR9rv6CrXqsqJ0meplOGvxUZhcWwuFOSE4MEsxHph3l9JeJJvc2ylpGxC5I3AJjm9ZdSuPdItgE111ZLUGruFGc$)  Please also include the statement “There was no additional external funding received for this study.” in your updated Funding Statement.

Please include your amended Funding Statement within your cover letter. We will change the online submission form on your behalf.

**We have included an amended funding statement in the edited manuscript and in the response letter above.**

3**.** Please include your full ethics statement in the ‘Methods’ section of your manuscript file. In your statement, please include the full name of the IRB or ethics committee who approved or waived your study, as well as whether or not you obtained informed written or verbal consent. If consent was waived for your study, please include this information in your statement as well.

**We have now included a full ethics statement in the Methods section, with the full name of the IRB and IRB number. We have clarified that participants provided written consent in order to participate.**

Reviewer #1: The authors correctly point to the fact that the experiments testing the effects of objectification have been typically underpowered, and that this may account for some inconsistent results. They also suggest that in these studies the use of covariates and of moderating variables might have been too ad-hoc, and "after the fact." Considering that the authors have no evidence of any of this, I found their statement in this regard (p. 11) unwarranted. This is a small detail, however.

**We have removed this statement about ad-hoc hypotheses as requested and have tempered our language on this issue throughout.**

To correct all this, they set out to conduct a well-powered, pre-registered experiment, with a three-level condition intended to test the effect of male gaze on cognitive performance of women. I truly applaud their systematic approach, the effort, and the fact that they pre-registered the study and shared all the data. The experiment, however, suffers from three major methodological flaws that make the results unpublishable.

1. Previous research, discussed by these authors, finds that the effects of an objectification manipulation is likely moderated by trait self-objectification. Objectification scholars clearly theorize this, as the authors themselves write on page 12 "As noted, self-objectification can be conceptualized as both a state and trait variable (Noll & Fredrickson, 1998), and therefore, in line with original theory, women who have the tendency to self-objectify could have a greater chance of being distracted by body monitoring." Yet, the authors decide NOT to take any trait measure of self-objectification. This alone could be the reason why they did not find effects of their manipulation (moderated by TSO, of course).

**The reviewer raises the concern that previous research on this topic shows the effect of state self-objectification on conscious attention is moderated by trait self-objectification (TSO). Although moderators can certainly be important to consider when testing theoretically derived hypotheses, our reading of the published literature on this topic does not lead us to conclude that TSO is an essential moderator when it comes to the effect of objectification on cognitive performance. For example, despite not including TSO as a moderator, Quinn et al. (2006) reported a significant main effect of objectification on a modified Stroop task performance and Gervais (2011) found women in the experimental condition performed worse on a timed math test relative to the control. Of those studies that do measure TSO or a similar trait-level variable, the results are mixed. Four studies (Tiggemann & Boundy, 2008; Hebl et al., 2004; Gapinski et al., 2003; Fredrickson et al., 1998) found no evidence that trait self-objectification moderates the effect of objectification on performance. Of these, one reports a significant main effect of objectification on performance with a cut-off *p* < .05(Hebl et al., 2004) and three do not (Tiggemann & Boundy, 2008; Gapinski et al., 2003; Fredrickson et al., 1998). In Study 1, Kahalon et al. (2018) found trait self-objectification moderated the effect of condition (i.e., receiving an appearance compliment) on math test scores, but did not replicate this finding in Study 2. Based on the means and standard deviations reported in Study 2, women in the experimental condition performed worse on a math test than those in the control condition by a Cohen’s d of 0.52 (a moderate-to-large effect), *without* accounting for TSO or previous math scores.**

**We noted two studies that found evidence of TSO or a similar trait-like variable as a potential moderator. Guizzo and Cadinu (2017) found no effect of condition on sustained attention to response task but reported internalized beauty ideals (a trait-level variable with similarities to TSO) as a significant moderator of this relationship. Gay and Castano (2010) used a manipulation that involved either a male or female experimenter video recording participant as they walked down a hallway, but did not include a no-video control in their study. They did not find a main effect of experimental condition (male vs. female experimenter) on a letter-number sequencing (LNS) task, LNS latency, or GMAT scores in Study 1 (*n* = 25) or Study 2 (*n* = 50). In Study 1, they did find a TSO by condition interaction on LNS latency, whereby participants with higher TSO performed worse in the condition with the male experimenter compared to the condition with the female experimenter (*p* = .02). The same TSO by condition interaction did not reach statistical significance in Study 2 (*p* = .07).**

**Overall, findings on TSO as a moderator of objectification’s impact on cognitive performance are mixed. We now address these findings more specifically in the discussion section, to help situate our findings in the current body of literature on this topic.**

**We designed our experiment to have sufficient power for the primary effect of interest (one-way ANOVA with 3 conditions) and found a moderate effect of experimental condition on body surveillance (i.e., state self-objectification). Although it is possible that participants with high TSO would be *even more* likely to experience state self-objectification (and thus be at greater risk of distraction during the task), we reasoned that participants who experience higher levels of state self-objectification would perform worse on the working memory task, even in the control condition. However, we found no significant associations between body surveillance and performance or latency on the working memory task for any condition. Nonetheless, we agree with the reviewer that it would be ideal if we had participants’ TSO scores for this study and address this as a limitation in our discussion section.**

2. The authors seem to model their manipulation after the study by Gay and Castano, in which the above-mentioned interaction was found. Yet, instead of having women in the male-gaze condition filmed by a man, they only tell them that the video might be later viewed by a man. Why the authors chose to water-down so much a manipulation that not only had worked in the past, but that it is also a direct operationalization of the theory of objectification and it has excellent ecological validity, is puzzling to me.

**We agree with the reviewer that the video recording manipulation used in Gay and Castano has excellent ecological validity and therefore, modeled our experiment on the premise that framing a woman’s body with a video camera from the neck down would simulate an objectifying gaze. However, instead of having a male vs. female experimenter video record participants, we opted to use an anticipated male gaze manipulation as in Calogero et al. (2004), as Calogero et al. found that anticipating a male gaze increased social physique anxiety (a construct that overlaps with body surveillance). Across two studies, Gay and Castano did not include a manipulation check (i.e., a measure of body surveillance/state self-objectification), so it is difficult to evaluate the extent to which their precise manipulation worked – especially since they found no main effects of their male vs. female experimenter manipulation. Gay and Castano did report some statistically significant effects for tests of two-way interactions and a three-way interaction (with TSO and item difficulty), but one interaction suggested that the female experimenter (vs. the male experimenter) had a more *negative* effect on latency. Given the very low sample sizes in Gay and Castano (25 participants in one study and 50 in another), it is difficult to know what to make of these interactions and the likelihood of false positive findings in these analyses appears high. Finally, unlike other studies that have used framing women’s bodies with a camera (Gay & Castano, 2010; Guizzo & Cadinu, 2017), we included a third condition with no video camera as a control, which we believe allows for a more direct test of our key hypothesis than using male vs. female experimenters as a manipulation, especially given evidence that female experimenters can also trigger self-objectification in women (Tiggemann & Boundy, 2008).**

**In our discussion section, we acknowledge that despite its efficacy in Calogero et al. (2004), it is possible that our anticipated male gaze manipulation was not strong enough and that this could explain why we did not see significantly different levels of body surveillance between our two experimental conditions (and found significant differences only between our experimental conditions and our control). Nonetheless, we observed no effect of condition on performance despite a moderate effect of the control versus the experimental conditions on body surveillance; we focus on results between experimental conditions and control rather than differences between the two experimental conditions.**

3. Previous research also shows that the task must be somewhat challenging for the effects of objectification to emerge. This is true, by the way, also for other social cognition research that does not deal with objectification: If it is too easy, you won't find the effect of the manipulation because participants can complete the task even when they are cognitively depleted. Yet, here too, the authors make the puzzling choice of selecting 20 very easy items: range 0-20, M = 16.33.

**We agree with the reviewer that if a task is too easy, participants may be able to successfully complete it even while cognitively depleted. In the case of our DV, the items required only basic arithmetic skills, but were cognitively taxing to solve because they had to be solved without pen and paper (i.e., in one’s head). In fact, solving mental math problems is commonly used in cognitive psychology as a manipulation that loads working memory (e.g., Ashcraft & Krause, 2007). We now include more discussion of the working memory task we used in the paper and provide citations to support the contention that these mental math problems were likely taxing to participants even if they were able to generate the correct answers. As evidence of this conclusion, the latency scores for these math problems had a substantial amount of variability.**

Reviewer #2: Thank you for the opportunity to review, “Brains over beauty: A pre-registered test of the effects of objectification on women’s cognitive performance.” The authors should be commended for conducting work that is pre-registered and adds clarity not only to an important tenant of objectification theory, but also to the literature that has provided mixed results in terms of the relation between women’s self-objectification and cognitive performance. I also would like to say that I thought this manuscript was very well-written and easy to follow, from the literature review to coverage of the results. Below I outline a few points that I believe could make this an even stronger manuscript.

I appreciated that the authors took time to outline all previous work conducted on self-objectification and cognitive performance. I found myself trying to trace citations to determine whether work that found significant effects was one in the same with work that was potentially underpowered. This made me think that including a table of all papers, with columns of all relevant pieces of information (e.g., manipulation, sample size, findings), could make for an easier examination of inconsistencies in past findings and possible reasons why (e.g., which seven of the studies were likely underpowered?).

**Thank you for this suggestion. We have now included a table that outlines the manipulation, dependent variable, and primary outcomes of previous work in this area.**

Given that the Guizzo paper assessed the closest concept to the current work - sustained attention - can the authors elaborate on what this task looked like? Could you also clarify that Guizzo’s moderator of internalized beauty ideals was an conceptualization of trait level self-objectification?

**Additional details about this paper are now included (see p. 9).**

My only true concern with this manuscript is that it seems the authors may have missed some key citations. For instance, Winn & Cornelius have a 2020 review of literature on the topic, which I think if referenced would provide further justification for the previous mixed results and need for clarity. Additionally, a few other studies came to my mind that examine the relation between self-objectification and cognitive performance (broadly defined): Baldissari & Andrighetto (2021), Aubrey & Gerding (2015), and the sexual harassment program of work from Gervais & Wiener. While the authors may have purposely not included these articles in their literature review, it’d be helpful to understand how they came to the conclusion that there are only 9 studies on the topic.

**We appreciate these suggestions for additional literature to include. We carefully considered the studies cited in the Winn and Cornelius (2020) review and have incorporated additional relevant studies from that review (including Aubrey & Gerding, 2015). We have not included Baldissari & Andrighetto (2021), as that paper examined a non-sexualized form of objectification that is less relevant to body monitoring. In general, we did not include literature focused on sexual harassment, consistent with the distinction in objectification theory research between body evaluation and unwanted explicit sexual advances (e.g., Kozee et. al., 2007).**

In the intro, the authors mention that much previous work includes covariates in analyses. While true, I think it is an inappropriate assumption that this is a post-hoc decision. It’s true we cannot know without pre-registration, but many of the covariates are supported by research or theory, so I feel like this language should be tempered. In the authors’ current stance, it seems as if they think this is always an unacceptable practice, but I’m not sure I agree.

**Thank you for this suggestion. We have removed this statement and have tempered our language throughout.**

Fredrickson & Roberts suggest that not all women will respond to instances of objectification in the same manner. This is also most likely true of experiences that prompt self-objectification, whether intra or interpersonally - for women high in trait self-objectification, they are likely to engage in higher levels of self-objectification than those lower in this trait. As a result, I was surprised when the authors revealed the correlations between state body surveillance and performance without taking condition into consideration. Because women in the control condition did not experience any environmental prompt to self-objectify, body surveillance measured among these participants would likely be akin to a trait level of self-objectification. Although not pre-registered, I would like to see these additional analyses at least in a footnote.

**As requested, we have added correlations separated by condition between body surveillance, response accuracy, and response speed to the results section.**

One issue I have with how researchers discuss manipulations involving objectification is a lack of specificity. While many claim to manipulate self-objectification, they are actually manipulating interpersonal objectification, from which increased self-objectification levels ensue. I think when discussing the manipulations used, this paper (and the literature more broadly) may benefit from a bit more specificity here in terms of what is truly being manipulated.

**We have added notes in the table of previous studies indicating whether the manipulation was focused on manipulating state self-objectification or focused on manipulated interpersonal objectification, which can result in increased state self-objectification. We hope this indicator and the description of the manipulation in the table can help clarify which manipulations were used in previous studies.**

Within the discussion, I’d like to see more take-away points from the current work. Specifically, while the authors contrast their findings with those of past work, how do these findings inform objectification theory? Moreover, beyond calls for replication and power analyses, do these findings have implications for how objectification researchers conduct their work (e.g., in a literature with such varied ways of manipulating objectification, are the two manipulations in the current work interchangeable?)?

**We thank the reviewer for this suggestion. We have substantially reworked the discussion sections to make the takeaway points clearer. We now discuss how our work contrasts with previous work in terms of objectification manipulation and working memory task. We urge future researchers to consider the differences between interpersonal and non-interpersonal objectification manipulations and highlight the theoretical reasons we think these manipulations are not interchangeable. We also include additional discussion regarding potential pathways for future research in this area, including women’s potential cognitive burden while being video conferencing.**

Smaller details:

I felt the abstract could have used a bit more specificity as well as a bit more elaboration about the implication of the findings.

**We have included additional details in the abstract and discussed the implications of the range of manipulations and dependent variables used in this area of research.**

While the authors note that the sample in the current work may have differed from past samples in terms of math abilities, the current sample seems demographically similar to past samples in terms of age and race. I think this similarity is worth mentioning. I also think it’s worth mentioning in the introduction that affect was included because of previous findings.

**Thank you for these suggestions. We now clarify in the discussion section that our sample was similar in terms of age and race as used in previous studies on this topic. In the Introduction, we clarify that affect outcomes (affect and guilt/shame) were included based on findings from previous work.**

Because the authors critically analyzed sample sizes of past work, could you benefit future work by providing the number of participants per condition after exclusions?

**Thank you for this suggestion. We have added sample sizes per condition after exclusions to the text and to the tables.**

I hope this feedback is helpful to the authors and I look forward to seeing this paper in its final state!

Reviewer #3: The paper “Brains over Beauty” tested, using a more rigorous methodology than that used in previous research, whether the induction of state self-objectification interferes with women’s cognitive performance. The paper has several advantages over past research in that it (a) used a sufficiently powered sample, (b) examined two different types of manipulations to induce self-objectification, and (c) used a dependent variable that is unlikely to be influenced by stereotype threat (which could be an alternative explanation to the effect(s) observed in several of the previous studies). Because of these strengths, and because finding out what *does* not work despite being theoretically plausible is highly important for scientific advancement (see Eronen & Bringmann, 2021), I think that the paper makes an important contribution to the literature on women’s sexual objectification. Nevertheless, I identified several weaknesses, which I list below.

**We appreciate the reviewer pointing to recent discussions about the strengths of null results and now incorporate arguments made by Eronen and Bringmann (2021) in our discussion.**

1. In my reading of objectification theory, the original 1997 paper by Fredrickson and Roberts aimed to explain (through the concept of self-objectification) why women experience higher rates of unipolar depression, eating disorders, and sexual dysfunctions as compared to men. The idea that self-objectification should interfere with cognitive performance, tested in the 1998 paper by Fredrickson et al., is an extension of the original theory (as put forward in the 1997 paper). I think that the literature review in the present paper should reflect this (unless the authors have a different view on how objectification theory evolved, and if so – perhaps they can explain their view, at least in the response letter).

**Our perspective is that the original 1997 paper laid the foundation for the claim that self-objectification could interfere with cognitive performance, arguing that "significant portions of women's conscious attention can often be usurped by concerns related to real or imagined, present or anticipated, surveyors of their physical appearance." The original paper also mentioned these types of cognitive effects when discussing how self-objectification can inhibit women’s movement: “These fits and starts apparent in women's movements may also affect women's mental concentration.” However, the reviewer makes a good point that this idea was more directly elaborated upon in Fredrickson et al. (1998). We have now updated the wording in the literature review to more accurately reflect this.**

2. When you discuss the research by Quinn et al. (2006) please note that there is a major flaw in how the DV (performance in a Stroop test) was calculated in this study (instead of looking at participants’ interference score, the authors looked at the overall reaction times without comparing congruent vs. incongruent trials – which is NOT how performance in a Stroop test should be calculated).

**We thank the reviewer for their careful reading of the literature and have clarified in the manuscript that Quinn et al. (2006) employed a modified Stroop task.**

3. I think that it will be very helpful to the readers if you could add a table summarizing the main findings of experimental research on the effects of state self-objectification (SSO) on cognitive performance. That is, for each research mention (in a separate column) how was SSO manipulated? what was the DV? what was the main finding(s)? was the study sufficiently powered? This information appears in the text, but it will be much more convenient for readers to have it all briefly concentrated in one place (in addition to the longer description that currently appears in the text).

**Thank you for this suggestion. We have now included a table that outlines the manipulation, dependent variable, and primary outcomes of previous work.**

4. The term “stereotype threat” is mentioned but not defined. I suggest briefly explaining what it means (as you cannot assume that all readers are familiar with this literature).

**We now provide a brief definition of stereotype threat in the manuscript.**

5. To the best of my knowledge, it is recommended to quantify the evidence in favor of the null hypothesis (which seems to be supported in the present study) using Bayesian hypothesis testing (e.g., Wagenmakers et al., 2018).

**Thank you for the suggestion and resource. We now include calculated Bayes factors in the text and in the supplementary materials online.**

6. In p. 18 there is a link to the test that participants had to solve, but it took me some time to find in which document it is located (because the link brings the reader to a list of 9 documents). So, you can say something like “see here (in the document titled MATH appendixes) for list of problems”.

**We have added this clarification to the manuscript.**

7. I wonder whether the manipulation in the “male gaze” condition could be strengthened by leading participants to believe that they are going to meet with a man who saw their video (taped from the neck down).

**We now include additional discussion regarding ways the male gaze condition could have been strengthened, including whether the woman thought she would interact with the man who viewed her video.**

8. I think that the fact that the authors used a simple (rather than a difficult) math task is an advantage of the present study. In my understanding, there is no theoretical basis to the prediction that the effects of SSO should appear for difficult rather than simple tasks. In other words, I don’t agree with the authors’ suggestion, in p. 27 in the GD, that “objectification may have a greater impact on difficult compared to easy tasks,” and that this possibility should be tested in future research. The use of a simple math task, as done by the authors, is correct. However, I do think that a measure of participants’ pre-existing math ability/performance or at least their math identification should have been included and controlled for. I understand that the random assignment to experimental conditions should “take care” of any pre-existing differences in math ability. Nevertheless, the results would be more convincing if the authors could show that they persist even when controlling for pre-existing differences in math performance and/or identification. This is because there are huge interpersonal differences in these variables, which can create a lot of “statistical noise”.

**We have added text to the discussion about the limitations of not controlling for previous math scores or math identification.**

9. In p. 28, you refer to the Fredrickson et al.’s (1998) study as if participants were “completing an advanced math test in a room alone while wearing a bathing suit”. To the best of my memory, participants in this study first tried on a bathing suit in front of a mirror, but then completed the math test wearing their regular clothes. Please double check it to verify that the information is correct.

**Thank you for your close reading of the manuscript and the suggestion to verify this information. We have carefully reviewed Fredrickson et al.’s (1998) study as well as the other studies that use the swimsuit vs. sweater manipulation. We can confirm they report in their procedure section that participants wore the swimsuit or sweater while completing the math test and additional outcome measures.**

10. In the GD, you discuss trait self-objectification (TSO) as a potential moderator. I think that not testing for moderation by TSO is a major limitation of the present study. The authors explain the choice not to measure TSO prior to the experimental manipulation in that they didn’t want to prime this concept. I agree with this explanation, but I think that at the very least they should mention that future studies can overcome this problem by splitting the study into two parts, such that in the first part the potential moderators (including TSO) are measured, and in the second part (which can be held one week later, or so) the experimental manipulation is induced and the DVs are measured (as done by Kahalon et al., 2018).

**We have included additional discussion about the role of TSO as a moderator in previous work and our reasoning for not including it as a moderator in the current study. We have added the suggestion that future research could separate the study into two parts to avoid demand characteristics when measuring TSO or a similar trait-like variable.**

11. Another potential moderator that can be proposed in the GD (to be tested in the future) is women’s enjoyment of their sexualization (e.g., Liss et al., 2011). It makes sense the negative effects on mood are observed for women who are low on this measure, but not for women who enjoy being admired by men.

**Thank you for the suggestion. We have added women’s enjoyment of their sexualization as a potential avenue for future study in the Individual differences section of the discussion.**
