# Supplementary material for: Brains over beauty: A preregistered test of the effects of objectification on women’s cognitive performance
Source: PLoS One. 2023 Sep 21;18(9):e0291853. doi: 10.1371/journal.pone.0291853 (PMC10513279; doi:10.1371/journal.pone.0291853)
Supplement: Attachment — Submitted filename: R2 Brains Over Beauty Reviewer Response.docx [file pone.0291853.s002.docx]

**Detailed Responses to Reviewer Comments**

**1. Reviewer 1 insists that trait self-objectification (TSO) is an essential moderator, and that this study is not publishable because we did not assess TSO. Reviewer 1 writes, “Previous research, discussed by these authors, finds that the effects of an objectification manipulation is likely moderated by trait self-objectification. Objectification scholars clearly theorize this.”**

We fundamentally disagree with Reviewer 1’s claim that Objectification Theory makes the prediction that the effects of interpersonal objectification rely on an interaction with trait-level objectification. A careful, line-by-line reading of Fredrickson and Roberts’ (1997) Objectification Theory reveals no such claim. Indeed, as we thoroughly review in our manuscript, many authors have found cognitive effects of objectification manipulations without considering an interaction with TSO (pp. 36-38). It is clear that many scientists view these effects as robust enough that they do not require TSO as a covariate in order to be measurable. This issue is far from settled in the scientific literature.

In our manuscript, we carefully outline the literature concerning the role of TSO in objectification’s effect on performance, for example, noting, “Four studies [2,14,19,23] found no evidence that trait self-objectification moderates the effect of objectification on performance. Of these, one reported a significant main effect of objectification on performance using the cut-off *p* < .05 [23], and three did not [2,14,19]. Kahalon et al. (Study 1, [24]) found trait self-objectification moderated the effect of condition (i.e., receiving an appearance compliment) on math test scores, but did not replicate this finding in the paper’s second study.”(p.37) In summary, some previous studies found that objectification manipulations increase self-objectification and impair cognitive performance regardless of trait self-objectification. Other studies have only been able to show these effects when using trait self-objectification as a moderator. Still others found no evidence for trait self-objectification as a moderator. In other words, based on empirical findings, it is not clear that researchers must control for trait-level self-objectification to see the effects of objectification manipulations. We have updated this section of the paper to include the recent study by Savage and Couture Bue (2023), which also found TSO did not moderate the relationship between state self-objectification and cognitive performance.

**In response to this revision, Reviewer 1 wrote, “Such a reading is inaccurate. The fact that the authors go on to detail the studies that have and have not looked at the moderating effects of TSO does not add anything to what I think is an insufficient answer…Especially since they model their study after the study by Gay and Castano, which finds the interaction effect that is predicted by the objectification theory.”**

First, we did not model our study on Gay and Castano. Further, as we noted in our revision letter, the role of TSO as a moderator in Gay and Castano was inconsistent across two studies. In Study 1 (*n* = 25), the authors did find a TSO by condition interaction on letter-number sequencing (LNS) latency, whereby participants with higher TSO performed worse in the condition with the male experimenter compared to the condition with the female experimenter (*p* = .02). The same TSO by condition interaction did not reach statistical significance in Study 2 with 50 participants (*p* = .07).

**2. Reviewer 1 remains concerned about the nature of the manipulation in our study. In the first round of revisions, Reviewer 1 wrote, “The authors seem to model their manipulation after the study by Gay and Castano, in which the above-mentioned interaction was found. Yet, instead of having women in the male-gaze condition filmed by a man, they only tell them that the video might be later viewed by a man. Why the authors chose to water-down so much a manipulation that not only had worked in the past, but that it is also a direct operationalization of the theory of objectification and it has excellent ecological validity, is puzzling to me.”**

It is true that multiple elements of our methodology differ from those employed by Gay and Castano. Our paper is by no means an attempt at a direct replication of this specific study and at no point have we claimed that it is. To help clarify, we have added substantial detail to our paper explaining how and why we chose the manipulations we did (p.14-15).

Though Reviewer 1 praises the strength of the manipulation of using a male vs. female experimenter (as in Gay & Castano), we do not find the results of the Gay and Castano paper to be a persuasive demonstration of the superiority of this particular method of manipulating interpersonal objectification. Gay and Castano did not include a manipulation check and in both studies, condition had no significant effects on two of the three DVs (even when TSO and difficulty were taken into account). Without more evidence, our confidence that the male vs. female experimenter manipulation effectively increased women’s state self-objectification is tempered.

Reviewer 1 suggests that we modeled our manipulation after the one used in Gay and Castano, however, in Gay and Castano participants were video recorded (by either a male or female experimenter) from the neck-down while walking up and down a hallway for 2 minutes. The participants then watched video recording in its entirety before completing a LNS task on a computer and a GMAT math test. In our study, participants were video recorded from the neck-down while simultaneously completing the cognitive task (an attention task modeled after a different section of the WAIS, not the LNS). Participants did not review the video at any point during the study.

We based the design of our studies on a thorough review of this body of literature, attempting to capitalize on the strengths of different approaches in different papers. We are unclear as to why Reviewer 1 is so highly focused on Gay and Castano’s manipulation, despite the  methodologically diverse body of literature on this topic.

Nonetheless, we took care to note the reviewer’s concerns in our response and in the revised discussion section (p. 31). From our response to the previous round of reviews: “In our discussion section, we acknowledge that despite its efficacy in Calogero et al. (2004), it is possible the anticipated male gaze manipulation was not strong enough and that this could explain why we did not see significantly different levels of body surveillance between our two experimental conditions (and found significant differences only between our experimental conditions and our control). Nonetheless, we observed no effect of condition on performance despite a moderate effect of the control versus the experimental conditions on body surveillance; we focus on results between experimental conditions and control rather than differences between the two experimental conditions.”

**3. Reviewer 1 suggests that item difficulty should have been taken into account in analyses. Reviewer 1 writes, “In this [sic] experiments the authors, who allegedly aim to provide a more sound test than what was done in previous experiments, not only do not vary item difficulty, but chose only simple ones. Puzzling.”**

We explained this choice of outcome measure in the manuscript (see pages 33-34)  and in responses to the first round of requested revisions. In our manuscript we note:

 “The simplicity of the problems was also intended to avoid stereotype threat effects, which are more likely to emerge on difficult tasks (for a review see Pennington et al.[36]). This was especially important as stereotype threat effects have been raised as an alternate explanation for women’s reduced performance on advanced math tests in objectification studies[2,22,24]” (p. 33).“Even simple arithmetic operations require substantial working memory and could be disrupted by other cognitive demands[65], such as body monitoring. Moreover, math problems are frequently employed as distractor tasks in experiments because they are cognitively tasking and load working memory (e.g.,[66])” (p. 34).

“Consistent with this goal, our results showed substantial variability in the time taken to complete the task, even among participants with perfect accuracy” (p. 34).

**Reviewer 1 additionally responded in the second round of reviews, “In fact, in the study by Gay and Castano, this was done in both experiments, and in both experiments the pattern of results indicates clearly that difficulty needs to be taken into consideration.”**

Again, we strongly disagree with the characterization that our study uses, or was designed to imitate, the methodology used in Gay and Castano. Nonetheless, we carefully reviewed Gay and Castano’s results in terms of item difficulty. Gay and Castano used a Letter Number Sequencing (LNS) task (accuracy and latency) and an advanced math test as DVs. It is unclear in the paper whether item difficulty was taken into account when analyzing the math test. Regardless, in Study 1, the authors report no statistically significant main effects or interactions in terms of the math test, and drop this DV from Study 2. The LNS task used in the paper is somewhat more similar to the DV employed in our study. Gay and Castano qualified their two-way interaction of TSO (high, low) and condition (male experimenter, female experimenter) with a continuous measure of item difficulty on the LNS task. In Studies 1 (n = 25) and 2 (n = 50), the authors found that for difficult items, participants with high TSO took longer in the male versus female experimenter condition. However, in Study 1 the same three-way interaction revealed that for difficult items, participants with low TSO were slower after being videoed by a female versus male experimenter. It is not clear why participants with low trait self-objectification would perform worse on difficult (versus easy) items after being videoed by a female (the “low” objectification condition) versus male experimenter (the “high” objectification condition). In terms of LNS performance, the authors find a TSO by difficulty interaction in Study 1, but no significant two- or three-way interactions in Study 2. Especially without a control condition, these results are difficult to interpret with confidence. Further, our confidence in the strength of statistically significant results from three-way interactions with very small sample sizes remains limited. Additionally, virtually no published papers on the cognitive effects of interpersonal objectification have examined item difficulty in this way. It is far from the norm with respect to empirical or theoretical approaches in this literature.

In sum, Reviewer 1 seems to argue that interpersonal objectification will have cognitive effects on women only if a) their TSO is taken into account and b) item difficulty is taken into account. As evident in Table 1 of our paper (which provides a summary of published studies on this topic, p.10-12), this does not appear to be true.
